# Side-leakage of face mask

**DOI:** 10.1140/epje/s10189-021-00081-2

**Published:** 2021-06-05

**Authors:** B. N. J. Persson

**Affiliations:** 1 PGI-1, FZ Jülich, Jülich, Germany; 2 Multiscale Consulting, Wolfshovener str. 2, 52428 Jülich, Germany

## Abstract

**Abstract:**

Face masks are used to trap particles (or fluid drops) in a porous material (filter) in order to avoid or reduce the transfer of particles between the human lungs (or mouth and nose) and the external environment. The air exchange between the lungs and the environment is assumed to occur through the face mask filter. However, if the resistance to air flow through the filter is high some air (and accompanied particles) will leak through the filter-skin interface. In this paper I will present a model study of the side-leakage problem.

**Graphicabstract:**

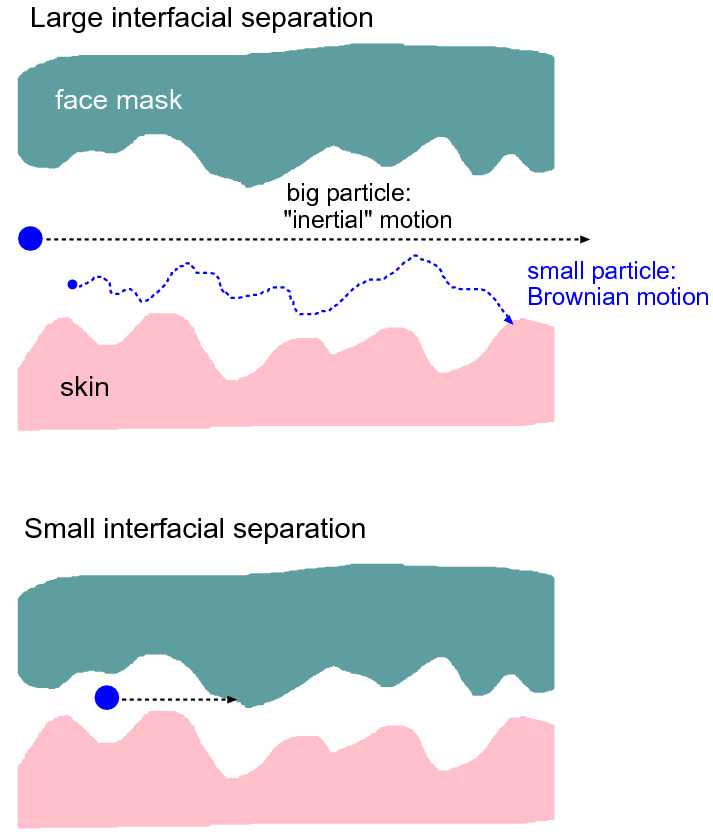

## Introduction

Face masks are used to trap particles in a porous material (filter) in order to avoid or reduce the transfer of particles between the human lungs and the external environment [1–3]. The filter usually consists of a sheet of randomly arranged fibers made from a polymer, e.g. polyethylene. The effectiveness of the filter will increase with increasing thickness of the filter and with decreasing size of the open channels through the filter. However, increasing the effectiveness of the filter will in general increase the resistance to the air flow through the filter, which may result in uncomfortable breathing experience, or side leakage of air between the skin and the filter surface. Thus, a recent report demonstrates the potential risk of increased face-to-mask seal leakage when N95 filtering facepiece respirators (N95 FFR) are covered by surgical, cloth, or medical masks [4]. In this paper I present a more detailed model study of the side-leakage problem. This problem was studied experimentally in Ref. [2] for dried sodium-chloride aerosols, and theoretically in Ref. [3] using fluid dynamics. For other recent studies, see [5–8].

## Theory

**Fig. 1:**
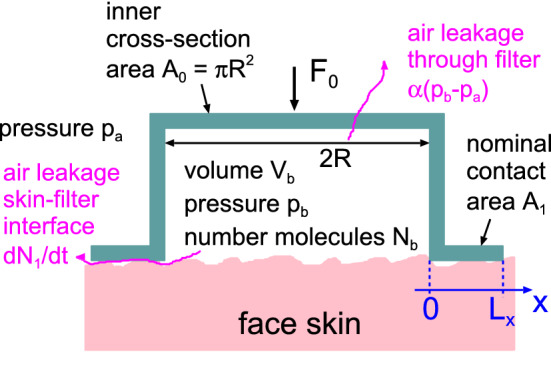
Model used for studying the air flow for a face mask. The air can flow through the filter at a number flow rate $\alpha ' (p_{\mathrm{b}} - p_{\mathrm{a}})$ or at the interface between the skin and the face mask at a number flow rate $\mathrm{d}N_1/\mathrm{d}t=\dot{N}_1$. The number of air molecules $N_{\mathrm{b}}$ and the air pressure $p_{\mathrm{b}}$ in the volume $V_{\mathrm{b}}$ between the face mask and the face varies in time due to the breathing act

We consider the simplest (idealized) case where the face mask makes contact with the skin over a circular annuls (radius *R*) of width $L_x$ in the radial (air leakage) direction and $L_y=2\pi R$ in the orthogonal angular direction (see Fig. 1). We assume the nominal contact pressure is constant in the nominal contact area $A_1 = L_x L_y$. The air volume between the face and the face mask is denoted by $V_{\mathrm{b}}$ and is assumed to be constant. The face mask is pushed against the skin by a force $F_0$ given by the extension of the rubber band, which is used to attach the face mask to the head, and with the force $A_0 (p_{\mathrm{b}}-p_{\mathrm{a}})$ due to the air pressure difference between inside and outside the mask. The spring contact pressure $p_{\mathrm{s}} = F_0/A_1$. The nominal contact pressure in the area $A_1$ is

1 $$\begin{aligned} p=p_{\mathrm{s}}-\beta (p_{\mathrm{b}}-p_{\mathrm{a}}), \end{aligned}$$

where

$$\begin{aligned} \beta =A_0/A_1+\beta ', \end{aligned}$$

where $p_{\mathrm{b}}$ is the air pressure inside the face mask and $p_{\mathrm{a}}$ the air pressure outside, which is assumed to be constant and equal to 1 atm ($1 \ \mathrm{bar}$). The factor $\beta '$ is a number between 0.5 and 1 which depends on how the air pressure change from $p_{\mathrm{b}}$ at $x=0$ to $p_{\mathrm{a}}$ at $x=L_x$ (see Fig. 1). Since $A_0/A_1 = \pi R^2/(2 \pi R L_x) = R/2L_x$ we have $\beta = R/2L_x + \beta '$. For N95 masks the contact width $L_x$ is relatively small, so that $R/2L_x>> 1$ and $\beta '$ is less important.

The number of air molecules $N_b(t)$ inside the face mask satisfies

2 $$\begin{aligned} \dot{N}_b = \dot{N}(t) - \alpha ' (p_{\mathrm{b}}-p_{\mathrm{a}}) - \dot{N}_1 \end{aligned}$$

where $\dot{N}$ is the number of air molecules entering the volume $V_b$ from the lungs, and $\alpha ' (p_{\mathrm{b}}-p_a)$ the number of air molecules leaking though the face mask filter. $\dot{N}_1$ is the number of air molecules leaking between the skin and the face mask, which depends on the nominal contact pressure *p* and the pressure difference $\Delta p = p_{\mathrm{b}}-p_{\mathrm{a}}$ between inside and outside. We will assume the ideal gas law so that

3 $$\begin{aligned} p_{\mathrm{b}} V_{\mathrm{b}} = N_{\mathrm{b}} k_{\mathrm{B}} T \end{aligned}$$

The leakrate $\dot{N}_1$ is given approximately by [9, 10]

4 $$\begin{aligned} \dot{N}_1 = {1 \over 24} {L_y\over L_x} {(p_{\mathrm{b}}^2 -p_{\mathrm{a}}^2 )\over k_{\mathrm{B}}T} {u_{\mathrm{c}}^3 \over \eta } \end{aligned}$$

Here $k_{\mathrm{B}}T$ is the thermal energy ($k_{\mathrm{B}}$ is the Boltzmann constant and *T* the absolute temperature), and $u_{\mathrm{c}}$ is an effective surface separation which we determine using the Persson contact mechanics theory [11–13] and the Bruggeman effective medium theory as described elsewhere [14–17].

The gas viscosity

$$\begin{aligned} \eta = {1\over 3} mn{\bar{v}} \lambda , \end{aligned}$$

where *n* is the gas number density and $\lambda$ the mean free path due to collisions between gas molecules. Note that $\lambda \sim 1/n$, so the viscosity $\eta$ is independent of the gas number density. Equations (1)–(4) are 4 equations for the 4 unknown quantities, *p*, $p_{\mathrm{b}}$, $N_{\mathrm{b}}$ and $N_1$.

We denote the resistance to air flow through the face mask filter by $1/\alpha$, where the air flow conductance $\alpha$ is defined by

5 $$\begin{aligned} {\mathrm{d} V \over \mathrm{d}t}= \alpha (p_{\mathrm{b}}-p_{\mathrm{a}}) \end{aligned}$$

where $\dot{V} = \mathrm{d} V/\mathrm{d}t$ is the volume of air of atmospheric pressure passing through the face mask filter per unit time given the pressure difference $\Delta p = p_{\mathrm{b}}-p_{\mathrm{a}}$ between inside and outside the face mask. Using the ideal gas law we have

6 $$\begin{aligned} p_{\mathrm{a}} \dot{V} = \dot{N} k_{\mathrm{B}} T = \alpha ' (p_{\mathrm{b}}-p_{\mathrm{a}}) k_{\mathrm{B}} T \end{aligned}$$

so that $$\alpha = \alpha ' k_{\mathrm{B}} T/p_{\mathrm{a}}$$.

## Numerical results

We will assume that air molecules are injected and removed from the volume $V_{\mathrm{b}}$ by the breathing action in a periodic way so that

$$\begin{aligned} N (t) = N_0 \mathrm{sin}(\omega _0 t) \end{aligned}$$

corresponding to a volume of air (of atmospheric pressure) $V(t) = k_{\mathrm{B}}T N(t)/p_{\mathrm{a}}$,

7 $$\begin{aligned} V (t) = V_0 \mathrm{sin}(\omega _0 t) \end{aligned}$$

where $\omega _0 = 2 \pi /T$ where *T* is the period of breathing. We assume that at time $t=0$, $p_{\mathrm{b}} = p_{\mathrm{a}}$ and hence from (4), $\dot{N}_1 = 0$ for $t=0$.

Ref. [21] present the air flow conductance of several types of face masks. For the US N95 face mask for the air flow $\dot{V} = 85 \ \mathrm{liter/min}$ the pressure difference $p_{\mathrm{b}}-p_{\mathrm{a}}$ should be smaller than $343 \ \mathrm{Pa}$ during inhalation and $245 \ \mathrm{Pa}$ during exhalation (the two pressure drops may differ because of side leakage). If the leakage would be entirely through the face mask these two cases correspond to $\alpha \approx 0.25$ and $\alpha \approx 0.35 \ \mathrm{liter /min \cdot Pa}$. For the European FFP2 face mask slightly larger (minimum) flow conductance are required. Below we show results for $\alpha = 0.3$ and $0.15 \ \mathrm{liter /min \cdot Pa}$, where the smaller value may reflect a N95 face mask contaminated by particles which block air flow channels.

In the numerical study we use $T=5 \ \mathrm{s}$ and $V_0 = 0.5 \ \mathrm{liter}$, corresponding to an air volume $1 \ \mathrm{liter}$ oscillating between the lungs and the outside of the lungs. We use the spring force $F_0=3 \ \mathrm{N}$ as measured for a N95 face mask on my own head. The face mask is assumed to make (nominal) contact with the skin over a circular strip of with $L_x=3 \ \mathrm{mm}$ in the (radial) air leakage direction and of length $L_y = 2 \pi R = 40 \ \mathrm{cm}$ is the orthogonal angular direction. The volume between the face and the face mask is assumed to be constant in time and equal to $0.1 \ \mathrm{liter}$. In reality the volume will fluctuate due to the oscillations in the air pressure $p_{\mathrm{b}}(t)$, but this effect is not significant.

To calculate the air side leakage it is necessary to know the surface roughness of the face mask and the skin in the nominal contact region, and we use the surface roughness measured on the wrist skin in Ref. [18, 19]. The roughness on the face skin may be different but we have not studied it, and I am not aware of any measured roughness power spectrum for the human face skin. It is known that the skin topography depends strongly on the gender, age, race of individual and humidity, and obtaining information about how the roughness at different length scale varies with these variables is an important future research topic. Such a study could be performed using silicone rubber replicas of the skin, which is known to be able to reproduce the roughness down to nanometer length scales [22]. We note that if the skin is covered by hair (beard, poorly shaven or unshaven) this could effectively strongly increase the surface roughness and result in much larger leakage rates than predicted in the study below [23]. In this section we will assume that the face mask surface has no surface roughness. Including the surface roughness on the face mask surface will increase the side leakage.

**Fig. 2:**
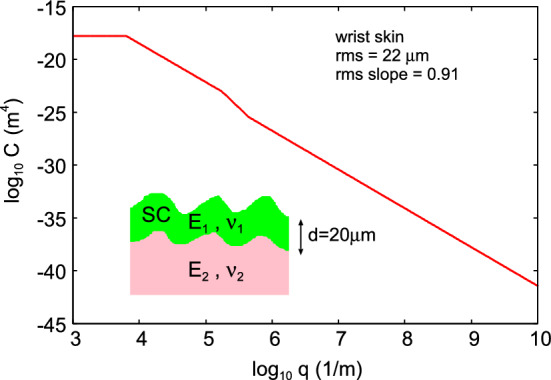
The surface roughness power spectrum as a function of the wavenumber (log-log scale) obtained from optical and AFM measurements of the surface topography of the wrist skin of a 49 years old man (from Ref. [18, 19]). The surface has the rms roughness amplitude $22 \ \mathrm{\mu m}$ and the rms slope 0.91. The inset shows the skin model used in the contact mechanics model calculations. The Young’s modulus and Poisson ratio of the top layer of the skin (stratum corneum, of thickness $d=20 \ \mathrm{\mu m}$) are $E_1=1 \ \mathrm{GPa}$ and $\nu _1=0.5$, while the material below the top layer has $E_2= 20 \ \mathrm{kPa}$ and $\nu _2=0.5$ (see [20] for information about the human skin elastic properties)

Figure 2 shows the surface roughness power spectrum as a function of the wavenumber (log-log scale) obtained from optical and AFM measurements of the surface topography of the wrist skin of a 49 year old man (from Ref. [18, 19]). The surface has the root-mean-square (rms) roughness amplitude $22 \ \mathrm{\mu m}$ and the rms slope 0.91. The inset shows the skin model used in the contact mechanics model calculations. The Young’s modulus and Poisson ratio of the top layer of the skin (stratum corneum, of thickness $d=20 \ \mathrm{\mu m}$) are $E_1=1 \ \mathrm{GPa}$ and $\nu _1=0.5$, while the material below the top layer has $E_2= 20 \ \mathrm{kPa}$ and $\nu _2=0.5$ (see [20] for information about the human skin elastic properties).

**Fig. 3:**
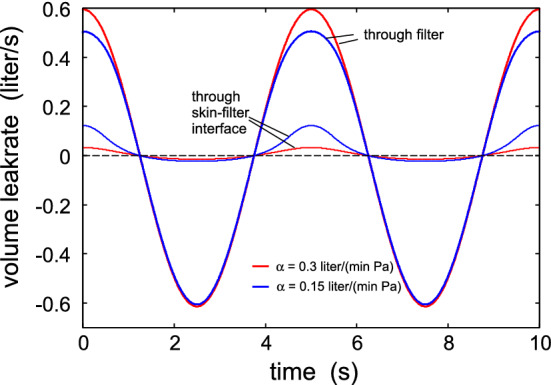
The air (of atmospheric pressure) volume leak rate in $\mathrm{liter/s}$ as a function of time during breathing at the period of $T=5 \ \mathrm{s}$ and the volume $V_0 = 0.5$ liter: $V(t)=V_0 \mathrm{sin}(\omega t)$ with $\omega = 2 \pi /T$. The red and blue curves are for the filter air flow conductance $\alpha = 0.3$ and $0.15 \ \mathrm{l/(min\cdot Pa)}$. The thick lines is the leakage through the filter and the thinner lines at the skin-filter interface

Figure 3 shows the air (of atmospheric pressure) volume leak rate in $\mathrm{liter/s}$ as a function of time during breathing. The red and blue curves are for the filter air flow conductance $\alpha = 0.3$ and $0.15 \ \mathrm{liter/(min\cdot Pa)}$. The thick lines is the leakage through the filter and the thinner lines at the skin-filter interface. Note that the side air leakage is larger during exhalation than during inhalation. This is due to the air pressure term $(p_{\mathrm{b}}-p_{\mathrm{a}})A_0$ which increases the force squeezing the face mask against the skin during inhalation, while it reduces the contact force during exhalation. For $\alpha = 0.3 \ \mathrm{liter/(min\cdot Pa)}$ for exhalation about $5\%$ of the air leaks between the skin and the face mask, while during inhalation only $2.6\%$ of the air side leaks. For $\alpha = 0.15 \ \mathrm{liter/(min\cdot Pa)}$ the corresponding numbers are $18\%$ and $4\%$.

**Fig. 4:**
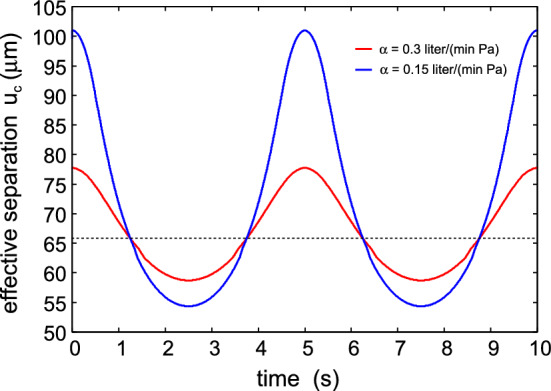
The effective surface separation as a function of time

**Fig. 5:**
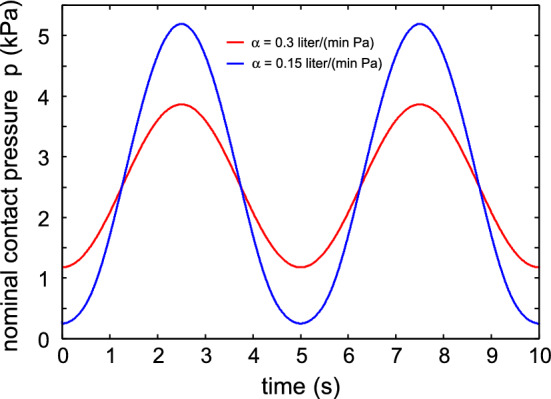
The nominal contact pressure in the filter-skin nominal contact area, as a function of time

**Fig. 6:**
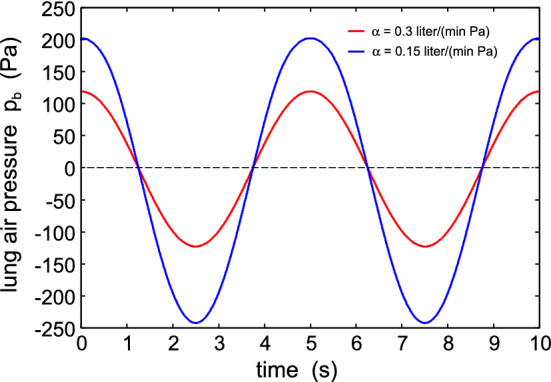
The air pressure (relative to the atmospheric pressure) in the lungs as a function of time

Figure 4 shows the effective surface separation $u_{\mathrm{c}}$ as a function of time. The dotted line indicates the effective surface separation when there is no pressure force, i.e., when $p_{\mathrm{a}}=p_{\mathrm{b}}$. As expected, during exhalation the surface separation increases while it decreases during inhalation. Similarly, the nominal contact pressure in the filter-skin nominal contact area decreases during exhalation and increases during inhalation as shown in Fig. 5. Figure 6 shows the air pressure (relative to the atmospheric pressure) in the face mask volume $V_{\mathrm{b}}$ as a function of time.

**Fig. 7:**
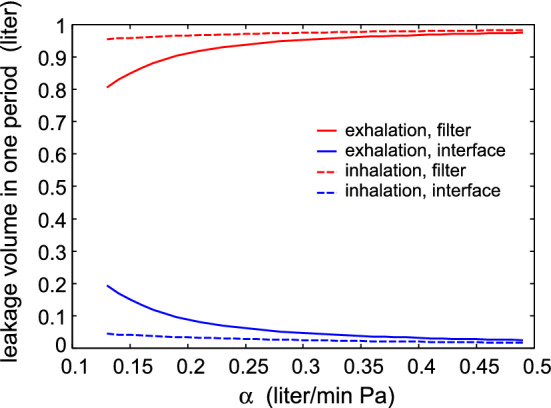
The volume of air passing through the filter (red lines) and through the skin-filter interface (blue lines) as a function of the filter air flow conductance. The solid lines is during exhalation and the dashed lines during inhalation

Finally, in Fig. 7 we show the volume of air passing through the filter (red lines) and through the skin-filter interface (blue lines) as a function of the filter air flow conductance. The solid lines is during exhalation and the dashed lines during inhalation. For small air filter conductance the side leakage is large during exhalation while during inhalation there is a much smaller side leakage.

## Discussion

The above study is very idealized as we have assumed a uniform contact pressure in the face mask-skin nominal contact area. We have also neglected the surface roughness on the face mask and treated the face mask as rigid when calculating the effective surface separation $u_{\mathrm{c}}$ which determines the leakrate.

**Fig. 8:**
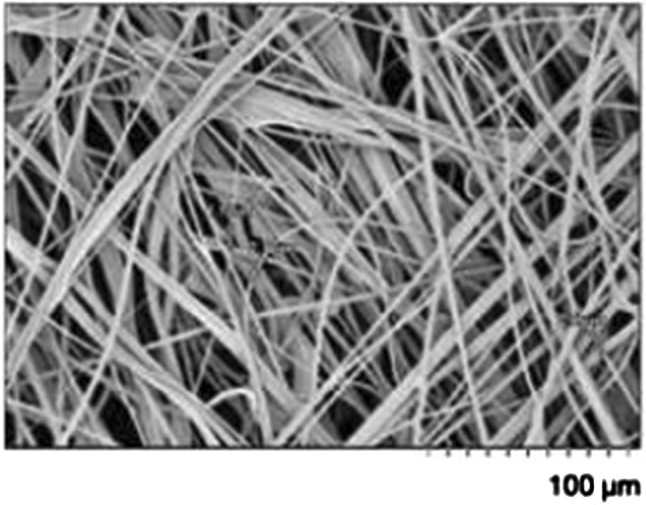
Scanning electron micrograph image of filter layer (non-woven polypropylene, melt blown). Adapted from Ref. [24]

The N95 face mask is built from non-woven polymer fibers, e.g., polypropylene, polyethylene or polyesters (see Fig. 8) [1]. (Recently it has been suggested to instead use polymer films with a periodic distribution of closely spaced nanoholes as face mask filters [27].) The polymers have a large elastic modulus (typically several GPa) but the fiber mat is macroscopically relative soft with an effective modulus in tension typically in the range $10-100 \ \mathrm{MPa}$. The fiber mat can be treated as an homogeneous material only on length scales larger than both the fiber width (or thickness), and the average distance between two nearby fiber segments which typically means distances of order $10 \ \mathrm{\mu m}$ or more. However, because of the low nominal contact pressure, the average and effective surface separation in the present case are determined by the longest wavelength surface roughness components so to a good approximation we can treat the fiber mat as a homogeneous material. Another problem is that the fiber mat cannot in general be treated as a isotropic elastic material but the effective modulus $E_z$ which determine the elongation (tension) normal to the film, which is important for the contact mechanics, may differ from the elastic modulus $E_x=E_y$ in tension within the film plane. However, if the fibers bind to each other where they touch each other, and if they are closely spaced, we expect the effective modulus $E_z$ to be similar to the modulus in tension within the plane.

**Fig. 9:**
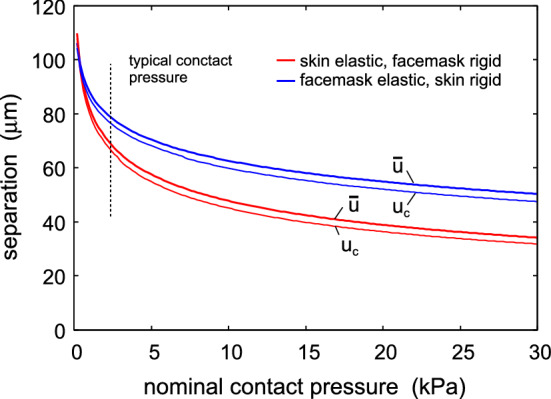
The average interfacial separation ${\bar{u}}$, and the separation $u_{\mathrm{c}}$ which determines the air leakage [see (4)], as a function of the nominal contact pressure. The red lines are the results assuming the face mask is rigid and the skin elastic (with layered elastic properties, see Fig. 2), and the blue lines is the result assuming the face mask elastic (with thickness $1 \ \mathrm{mm}$ and with $E=27.5 \ \mathrm{MPa}$ and $\nu =0.37$) and the skin rigid. Only the surface roughness of the skin (with the power spectrum shown in Fig. 2) is included in the calculations

We now show that including the macroscopic elastic properties of the face mask material, by using the modulus obtained in tension, will not result in any drastic change in the results presented above. To illustrate this, we show in Fig. 9 the average interfacial separation ${\bar{u}}$, and the separation $u_{\mathrm{c}}$ which determines the air leakage [see (4)], as a function of the nominal contact pressure. The red lines are the results assuming the face mask is rigid and the skin elastic (with layered elastic properties, see Fig. 2), and the blue lines is the result assuming the face mask elastic (with $E_x=27.5 \ \mathrm{MPa}$ and $\nu =0.37$, as measured for non-woven polypropylene in tension [28, 29]) and the skin rigid. In the latter case we have assumed the face mask has the thickness $1 \ \mathrm{mm}$, but practically the same result is obtained assuming infinite thickness. Only the surface roughness of the skin (with the power spectrum shown in Fig. 2) is included in the calculations.

**Fig. 10:**
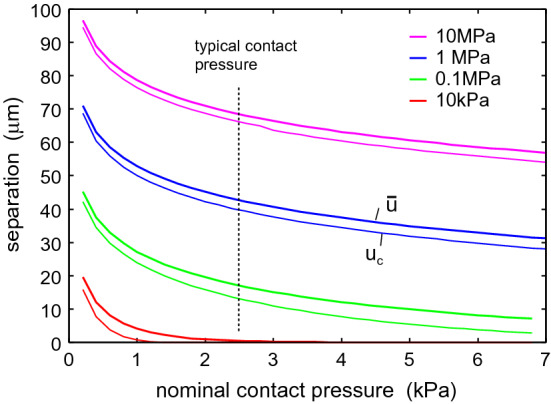
The average interfacial separation ${\bar{u}}$, and the separation $u_{\mathrm{c}}$ which determines the air leakage [see (4)], as a function of the nominal contact pressure. Results are shown for the elastic modulus $E=10$, 1, 0.1 and $0.01 \ \mathrm{MPa}$. In all cases the Poisson ratio $\nu =0.5$. Only the surface roughness of the skin (with the power spectrum shown in Fig.  2) is included in the calculations

**Fig. 11:**
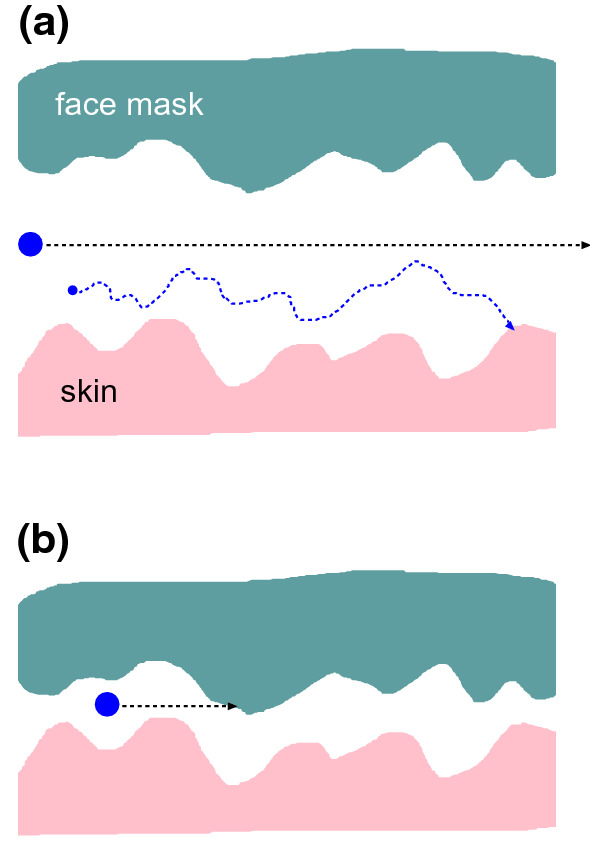
A large particle or droplet, due to its large inertia, will not be able to respond to the rapidly fluctuating (due to the surface roughness) air flow current and will hence move through the skin-filter nominal contact region on a nearly straight line. **a** If the average spacing between the surfaces in the air flow channels are much larger than the surface roughness amplitude then big particles may pass through the contact without collisions with the walls. **b** If the average spacing is of order, or smaller than, the surface roughness amplitude the big particle is likely to hit into the solid walls. A very small particle will perform Brownian motion in addition to drifting with the air flow. In this case if the Brownian motion amplitude is big enough the particle may hit into a wall even if the average wall separation is large as in (**a**)

We show below that in order to effective trap particles and droplets the surface separation in the nominal skin-face mask contact region should be much smaller than shown in Fig. 9. This can be realized if the rim of the face mask is covered by a strip of a soft material, e.g., weakly crosslinked Polydimethylsiloxan (PDMS). To illustrate which elastic modulus is necessary, in Fig. 10 we show the average interfacial separation ${\bar{u}}$, and the separation $u_{\mathrm{c}}$ which determines the air leakage [see (4)], as a function of the nominal contact pressure. Results are shown for the elastic modulus $E=10$, 1, 0.1 and $0.01 \ \mathrm{MPa}$. I all cases the Poisson ratio $\nu =0.5$. Only the surface roughness of the skin (with the power spectrum shown in Fig. 2) is included in the calculations. Note that for $E<10 \ \mathrm{kPa}$ the contact area percolate at a pressure below the average pressure in the skin-face mask contact region, so for such soft material no leakage of air or particles would be possible. In the calculations we have neglected adhesion which is important for the leakage only for very soft materials with $E<0.1 \ \mathrm{MPa}$ (see Appendix A). Thus including adhesion for the $E= 10 \ \mathrm{kPa}$ case in Fig. 10 would reduce ${\bar{u}}$ and $u_{\mathrm{c}}$.

**Fig. 12:**
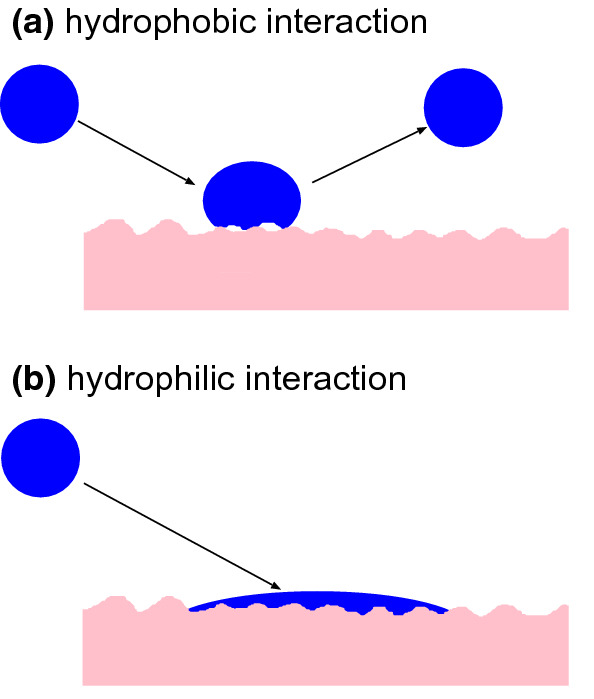
**a** If the fluid-solid interaction is hydrophobic, a liquid droplet hitting a solid may bounce off without transfer of fluid to the solid wall [25, 26]. **b** If the interaction is hydrophilic, the droplet may be adsorbed on the solid wall

**Fig. 13:**
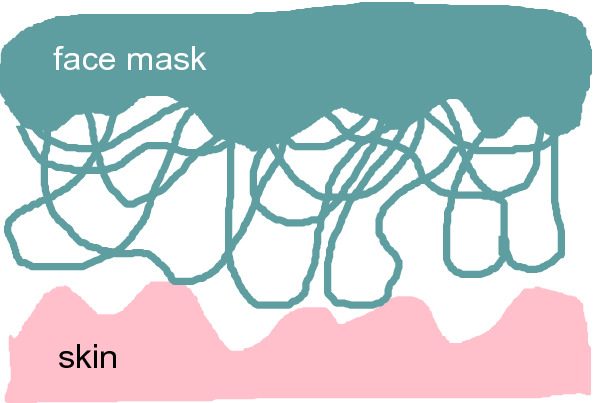
The N95 mask has a thin layer of fibers pointing away from the face mask surface which may help to trap particles in the side leakage air flow channel

A weakly crosslinked PDMS would be very sticky and perhaps uncomfortable to use and may be contaminated by dust particles. Another possibility would be to use an elastically soft hydrogel [30, 31] strip at the edge of the face mask, with a thickness of order a few mm. A proper chosen hydrogel may exhibit no or negligible adhesion to the human skin [32]. Hydrogels will dehydrate and becomes rigid in air, but perhaps the moisture in the air from the lungs is enough to keep it hydrated.

So far we have only considered the air flow problem. The question is now if particles in the air are able to follow the air flow into or out of the face mask volume $V_{\mathrm{b}}$. For the flow through the face mask filter this problem has been studied in detail both theoretically and experimentally. Several different mechanisms have been proposal which result in trapping of particles in the filter:

(A) Inertial impacting: Aerosol or dust particles typically $1 \ \mathrm{\mu m}$ or larger in size with enough inertia to prevent them from flowing around the fibers in the filtration layers slam into the mask material where they may adhere (but see below) and get filtered.
(B) Diffusion: Particles smaller than $1 \ \mathrm{\mu m}$, usually $0.1 \ \mathrm{\mu m}$ and smaller that are not subject to inertia undergo diffusion and become stuck to fibrous layers of the filter while undergoing Brownian motion around the tortuous porous matrix of the filter fiber.
(C) Electrostatic attraction: This mechanism employs electrocharged polymer or resin fibers that attract both large and small oppositely charged particles, or neutral particles via polarization (induced dipole) effects, and trap them. This effect depends on the distribution of positive and negative charges on the polymer surfaces (the total charge is likely to vanish so there must be an equal number of positive and negative charges on the polymer fibers) [33].

The critical or equivalent pore diameter (see Appendix B and Ref. [34]) in currently available N95 masks are around $300 \ \mathrm{nm}$ in size, while the SARS-CoV-2 virus is significantly smaller at 65 to $125 \ \mathrm{nm}$. However, the virus always travels attached to larger particles that are consistently snared by the filter. Thus, the virus usually attaches to water droplets or aerosols (i.e. really small droplets) that are generated by breathing, talking, coughing, etc. These consist of water, mucus protein and other biological material and are all of order or larger than $1 \ \mathrm{\mu m}$. Even if the particles were smaller than the N95 filter size, the erratic Brownian motion of particles that size and the electrostatic attraction generated by the mask means they would be consistently caught as well.

The fibers in face masks are usually made from a hydrophobic polymer in order to avoid the face mask absorb moisture from the air from the lungs. However, experiments have shown that a water droplet hitting a hydrophobic surface may bounce off which would result in a reduced trapping of fluid (aerosols) droplets (see Fig. 12) [25, 26]. Thus an interesting problem is to find out which water contact angle is optimal in order to avoid (or reduce) water absorption from the humid air from the lungs but still allow water droplets to become stuck to the fibers during impact from the air. Once stuck to a fiber, it is also important what happens to a respiratory droplet (e.g. evaporation of water) as this may effect the time period a trapped virus (or bacteria) is intact or alive [35]. This too will depend on the chemical nature (and the surface topography) of the fiber material.

The N95 and FFP2 face masks have thin layers of fibers pointing away from the face mask surfaces which may help to trap particles in the side leakage air flow channel (see Fig. 13).

Experiments have shown that N95 masks are actually best for particles either larger or smaller than the $300 \ \mathrm{nm}$ threshold. Thus N95 masks actually have that name because they are 95% efficient at stopping particles in their least efficient particle size range in this case those around $0.3 \ \mathrm{\mu m}$. Thus, particles smaller than $\sim 1 \ \mathrm{\mu m}$ perform erratic, zig-zagging Brownian motion with large enough amplitude to hit into a fiber, which greatly increases the chance they will be snared by the mask fibers.

The trapping mechanisms (A)-(C) are also relevant for trapping if particles in the air stream between the skin and the face mask. However, the effective separation between the skin and the face mask is much larger then the $0.3 \ \mathrm{\mu m}$ pore size in the face mask filter. Thus Fig. 4 shown that $u_{\mathrm{c}}$ is typically between $50-100 \ \mathrm{\mu m}$ which is several times bigger than the skin rms roughness amplitude (about $20 \ \mathrm{\mu m}$). Hence it is possible for micrometer sized particles to pass through the skin-face mask contact region as indicated in Fig. 11a. In order for the inertia effect trapping mechanism to be effective, one would need the average surface separation to be of order the rms surface roughness amplitude as indicated in Fig. 11b. Furthermore, the Brownian motion trapping mechanism (B) (see Fig. 11a) may be ineffective to trap small particles. Note that during a time *t* the mean square displacement, due to Brownian motion of a spherical particle (radius *R*), in one direction is given by [36]

5 $$\begin{aligned} \langle x^2 \rangle = {k_{\mathrm{B}}T t \over 3 \pi \eta R} \end{aligned}$$

The (average) air flow velocity *v* in the skin-face mask interfacial region is given by $\dot{V} = v L_y {\bar{u}}$ where $\dot{V}$ is the volume rate of air leakage at the interface and ${\bar{u}}$ the average surface separation. Using $\dot{V} =0.03 \ \mathrm{liter/s}$ and ${\bar{u}} = 70 \ \mathrm{\mu m}$ gives $v \approx 1 \ \mathrm{m/s}$. We assume the Brownian particle drift with the air stream so the time in the interfacial region will be $t = L_x/v$ or $t \approx 3\times 10^{-3} \ \mathrm{s}$ where we have used $L_x=3 \ \mathrm{mm}$. Using the air viscosity $\eta \approx 2\times 10^{-5} \ \mathrm{Pas}$ and $\langle x^2 \rangle \approx {\bar{u}}^2$ we get

$$\begin{aligned} R \approx {k_{\mathrm{B}}T t \over 3 \pi \eta {\bar{u}}^2} \approx 10^{-11} \ \mathrm{m} \end{aligned}$$

Thus, trapping of particles resulting from Brownian motion is negligible in the side leakage channel. This is different in the face mask filter, where the open channels may on the average have a diameter of order $1 \ \mathrm{\mu m}$ (and the most narrow constriction may be only $0.3 \ \mathrm{\mu m}$). This will enhance *R* by a factor $(70/1)^2 \approx 5000$ which will make Brownian motion important for particles smaller than $\sim 0.1 \ \mathrm{\mu m}$.

**Fig. 14:**
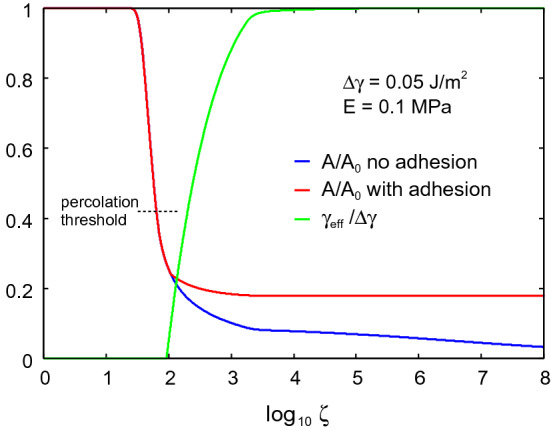
The relative contact area $A/A_0$ with (red line) and without (blue line) adhesion, and the effective interfacial binding energy $\gamma _{\mathrm{eff}}$ (or work of adhesion) (green line) as a function of the logarithm of the magnification. We have used the elastic modulus $E=0.1 \ \mathrm{MPa}$ and the work of adhesion for smooth surfaces $\Delta \gamma = 0.05 \ \mathrm{J/m^2}$. Note that the contact area percolate at a magnification where the adhesion does not manifest itself

## Summary and conclusion

We have studied a very simple model for the side leakage of face masks. We have assumed that the skin-face mask nominal contact pressure is the same everywhere in the nominal contact area, and neglected the surface roughness of the face mask surface. The calculations indicate that under normal conditions for the N95 or FFP2 face masks a few % of the air may leak through the skin-face mask interface. The average separation between the surfaces in the skin-face mask contact region is much larger than the effective pore size in the face mask filter which allow suspended particles in the air to enter or leave the face mask volume $V_{\mathrm{b}}$ during inhalation and exhalation.

## Figures and Tables

**Fig. 15:**
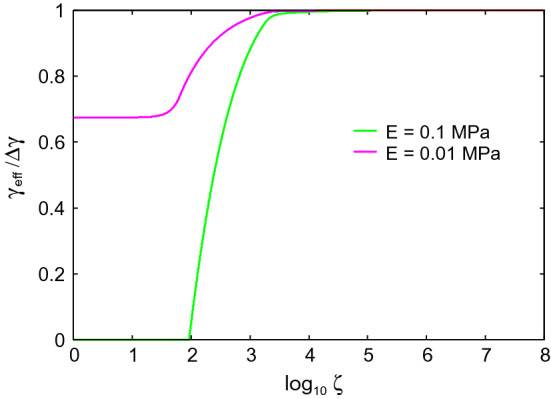
The effective interfacial binding energy $\gamma _{\mathrm{eff}}$ (or work of adhesion) as a function of the logarithm of the magnification. We have used the elastic modulus $E=0.1 \ \mathrm{MPa}$ (green line, from Fig. 14) and $E=0.01 \ \mathrm{MPa}$ (pink line), and the work of adhesion for smooth surfaces $\Delta \gamma = 0.05 \ \mathrm{J/m^2}$. Note that $\gamma _{\mathrm{eff}}$ vanish for $\zeta = 1$ when $E=0.1 \ \mathrm{MPa}$ while it is nonzero when $E=0.01 \ \mathrm{MPa}$. Thus only in the latter case will there be a finite pull-off force in the adiabatic (infinitely slowly) pull-off limit

## Data Availability

Data sharing is not applicable to this article as no new data were created or analyzed in this study.

## Appendix A: Role of adhesion

In calculating the air leakrate we have used the effective medium approach combined with the Persson contact mechanics theory for the probability distribution of surface separations. The basic contact mechanics picture (critical junction theory) which can be used to estimate the leak-rate of seals is as follows: Consider first a seal where the nominal contact area is a square. The seal separate a high-pressure gas on one side from a low pressure gas on the other side, with the pressure drop $\Delta P$. We consider the interface between the solids at increasing magnification $\zeta$. At the magnification $\zeta$ only roughness components with wavenumber $q<\zeta q_0$ can be observed, where $q_0$ is the smallest wavenumber. At low magnification we observe no surface roughness and it appears as if the contact is complete (see blue line in Fig. 14). Thus studying the interface only at this low magnification we would be tempted to conclude that the leak-rate vanishes. However, as we increase the magnification $\zeta$ we observe surface roughness and non-contact regions, so that the contact area $A(\zeta )$ is smaller than the nominal contact area $A_0 = A(1)$. As we increase the magnification further, we observe shorter wavelength roughness, and $A(\zeta )$ decreases further. For randomly rough surfaces, as a function of increasing magnification, when $A(\zeta )/A_0 \approx 0.42$ the non-contact area percolate [14], and the first open channel is observed, which allow fluid to flow from the high pressure side to the low pressure side. The percolating channel has a most narrow constriction over which most of the pressure drop $\Delta P$ occur. In the simplest picture one assume that the whole pressure drop $\Delta P$ occur over this *critical constriction*, and if it is approximated by a rectangular pore of height $u_{\mathrm{c}}$ much smaller than its width *w* (as predicted by contact mechanics theory), the leak rate can be approximated by Eq. (4). The height $u_{\mathrm{c}}$ of the critical constriction can be obtained using the Persson contact mechanics theory (see Ref. [11–13]).

When adhesion is included the average interfacial separation ${\bar{u}}$ and $u_{\mathrm{c}}$ decreases. However, the influence of adhesion on these quantities is in the present case negligible when the elastic modulus $E>0.1 \ \mathrm{MPa}$. This can be understood by studying how the contact are decreases with the magnification. Fig. 14 shows the relative contact area $A/A_0$ with (red line) and without (blue line) adhesion, and the effective interfacial binding energy $\gamma _{\mathrm{eff}}$ (or work of adhesion) (green line) as a function of the logarithm of the magnification [37, 38]. We have used the elastic modulus $E=0.1 \ \mathrm{MPa}$ and the work of adhesion for smooth surfaces $\Delta \gamma = 0.05 \ \mathrm{J/m^2}$ (as typical for adhesion involving PDMS). Note that the contact area percolate at a magnification where the adhesion does not manifest itself.

Figure 15 shows the effective interfacial binding energy $\gamma _{\mathrm{eff}} (\zeta )$ (or work of adhesion) as a function of the logarithm of the magnification $\zeta$. We have used the elastic modulus $E=0.1 \ \mathrm{MPa}$ (green line) and $E=0.01 \ \mathrm{MPa}$ (pink line), and the work of adhesion for smooth surfaces $\Delta \gamma = 0.05 \ \mathrm{J/m^2}$. Note that $\gamma _{\mathrm{eff}}(1)$ vanish when $E=0.1 \ \mathrm{MPa}$ while it is nonzero for when $E=0.01 \ \mathrm{MPa}$. For the latter case the calculation shows that the contact area percolate even when there is no external applied pressure, and no side leakage is possible. In addition, in this case, since $\gamma _{\mathrm{eff}}(1) > 0$, the pull-off force is finite even in the adiabatic (infinitely slowly) pull-off limit.

We note finally that for some hydrogel–countersurface systems it is possible to avoid adhesion [32], in particular at the low contact pressures of interest here. The effective repulsion in these systems is an entropy effect related to squeezing out water from the interface resulting in a higher concentration of the solvated ions in the water, which screen surface charges bound to the gel surface.

## Appendix B: Effective pore size

Filters made from non-woven polymer fibers have complex irregular air flow channels and no well defined pore size. For these systems one speak about equivalent pore diameter which can be determined using the bubble-point test as described in Ref. [34].

Consider all percolating open channels through the filter. For each channel there will be a most narrow constriction. The equivalent pore diameter is a length characterizing the width of the biggest of all these most narrow constrictions [34]. It can also be considered as the diameter of the critical constriction as introduced in the theory of seals developed in Ref. [15–17]. In this theory the system is studied at increasing magnification or resolution. At the lowest magnification (say naked eye) the fiber mat appears as a homogeneous material and no leakage would be expected at this magnification. As we increase the magnification we observe some “big” cavity regions but they do not percolate so even at this magnification one would not expect any leakage. When we increase the magnification even more (say using an optical microscope) one finally observe for the first time an open percolating path. The critical constriction is the most narrow constriction along the first percolating path which can be observed with increasing magnification. The equivalent pore diameter is roughly the diameter of a circular hole with the same cross section area as that of the critical constriction.
